# Differentiating Radiotherapy-Specific Distress From General Cancer Distress: Natural Language Processing Analysis of Patient Narratives

**DOI:** 10.2196/100874

**Published:** 2026-07-14

**Authors:** Yike Wang, Meiyan Ji, Xinghua Bai

**Affiliations:** 1 Department of Radiation Oncology, The First Hospital of China Medical University, No. 210, Baita 1st Street, Hunnan District, Shenyang, Liaoning, 110167, China, +86-1384001728

**Keywords:** psychosocial distress, psychosocial oncology, radiotherapy, patient experience, natural language processing, topic modeling

## Abstract

**Background:**

Psychological distress is common among patients with cancer, and it negatively impacts treatment adherence and quality of life. Radiotherapy, with its unique procedures, such as daily sessions and physical immobilization, may induce distress distinct from general cancer anxiety. However, existing screening tools cannot differentiate these distress sources. This study leverages online patient narratives and natural language processing to distinguish radiotherapy-specific distress from general cancer distress.

**Objective:**

This study aimed to systematically identify, differentiate, and compare the composition, structure, and emotional characteristics of general cancer distress and radiotherapy-specific distress through the analysis of large-scale online patient narratives.

**Methods:**

Using a retrospective observational design, we screened 52,831 relevant posts published between 2015 and 2025 on the online health community Reddit, ultimately including 9860 first-person patient narratives meeting the inclusion criteria. Content-based extraction revealed that the most frequently represented cancer types were head and neck (1576/6625, 23.8%), breast (1418/6625, 21.4%), and prostate (1000/6625, 15.1%), with the majority of posts written during active treatment (3587/5748, 62.4% of phase-identifiable posts). We used latent Dirichlet allocation thematic modeling for topic identification, supplemented by manual qualitative coding for thematic classification. Structural relationships between topics were analyzed via correlation heatmaps, while emotional polarity and discrete sentiments for both distress categories were quantitatively compared using VADER (Valence Aware Dictionary and Sentiment Reasoner) and RoBERTa models.

**Results:**

Radiotherapy-specific distress formed a thematically distinct and substantial domain, accounting for 50.4% (4969/9860) of all narratives, comparable to the proportion of 49.0% (4831/9860) attributed to general cancer distress. The remaining 0.6% (60/9860) was unclassifiable into either category. Thematic correlation analysis revealed that both categories exhibited high internal cohesion but weak intercategory associations, a pattern consistent with thematic differentiability between the 2 domains rather than a single undifferentiated construct. Sentiment analysis further revealed that radiotherapy-specific distress carried significantly stronger negative emotional intensity (Mann-Whitney *U* test, *P*<.001; rank-biserial correlation=0.34), with core emotions dominated by “fear” (2773/4969, 55.8%) and “anger/frustration” (1262/4969, 25.4%), whereas general cancer distress was more frequently expressed as “anxiety” (2183/4831, 45.2%) and “sadness” (1599/4831, 33.1%; *χ*² test, *P*<.001; Cramér V=0.22).

**Conclusions:**

This study provides exploratory evidence that radiotherapy-specific distress is thematically and emotionally differentiable from general cancer distress, suggesting it may constitute a distinct domain warranting targeted assessment and intervention strategies. Developing targeted assessment and care strategies addressing radiotherapy-specific challenges is essential for achieving truly patient-centered, individualized psychosocial support in oncology.

## Introduction

Psychological distress, a syndrome encompassing negative emotional responses, such as anxiety, depression, and existential concerns, is a common secondary diagnosis experienced by patients with cancer throughout their disease journey [1]. Extensive research has confirmed that significant psychological distress negatively correlates with treatment adherence, quality of life, and health outcomes [2-4]. Consequently, the precise identification and effective management of cancer-related psychological distress have become core components of high-quality holistic oncology care.

Among the various cancer treatment modalities, radiotherapy has the highest prevalence, with over half of patients receiving radiation therapy during their treatment course [5]. Although radiotherapy is widely recognized as a cornerstone of tumor control, its unique treatment patterns—including daily repeated sessions, treatment in relatively isolated environments, and physical constraints imposed by immobilization devices (eg, head-and-neck radiation masks)—may induce specific psychological stressors that are distinct from those experienced with other therapies [6]. Screening tools that are currently used in clinical practice, such as the distress thermometer [7], are effective in identifying the presence and intensity of distress at a macro level. However, these tools have limitations in distinguishing the subtle nuances of distress sources. A critical clinical question is whether patient-reported anxiety stems from generalized concerns about disease progression or from acute panic triggered by specific daily treatment situations, such as physical immobilization. These 2 distinct forms of distress may possess fundamentally different emotional textures and require inherently different intervention strategies.

Traditional research methods provide a crucial foundation for differentiating these distress sources, though each approach has inherent limitations [8,9]. While qualitative interviews offer rich, in-depth insights, they are typically constrained by small sample sizes, which can limit the generalizability of their conclusions. Conversely, large-scale questionnaire surveys may fail to capture unexpected, spontaneous, and unique patient concerns due to their predetermined frameworks. In recent years, the proliferation of online health communities has emerged as a significant source of data, offering a valuable opportunity to explore patients’ real-world experiences [10,11]. Within these platforms, a significant number of patients share their treatment journeys and inner feelings anonymously, thereby forming a vast repository of “patient-generated health data” with high ecological validity. The integration of natural language processing (NLP) technology facilitates the systematic analysis of unstructured text data. This approach supports the construction of a comprehensive landscape of patient psychological distress through a data-driven methodology, thereby enabling the dissection of its intricate internal structures.

This study aims to analyze large-scale online patient narrative texts using NLP methods to achieve the following four objectives: (1) identify and describe the core psychological distress themes expressed by patients with cancer undergoing radiotherapy; (2) categorize these themes into general cancer distress and radiotherapy-specific distress; (3) examine the internal consistency and intercategory differentiation patterns of this classification framework through interthematic correlation analysis; and (4) compare the differences in emotional intensity and discrete emotion types between these 2 categories. The study anticipates that its findings will provide empirical evidence for clinical practitioners—including nurses, psychologists, and radiation therapists—to facilitate the development of more targeted psychological intervention strategies aimed at enhancing the treatment experience and mental health outcomes of patients undergoing radiotherapy.

## Methods

### Study Design

This study used a retrospective observational design combining computational NLP with expert qualitative interpretation in a hybrid analytic framework. Specifically, unsupervised topic modeling was used for data-driven theme discovery, followed by researcher-driven qualitative interpretation for thematic labeling and classification. This hybrid approach leverages the scalability of computational methods while acknowledging that meaningful clinical interpretation requires domain expertise applied to algorithm outputs. The report adheres to the STROBE (Strengthening the Reporting of Observational Studies in Epidemiology) guidelines [12].

### Data Sources and Collection

The data for this study were obtained from Reddit, a prominent global online health community. A curated selection of subreddits with a high degree of relevance to cancer and radiation therapy was made, with a focus on r/cancer, r/RadiationTherapy, and other communities concentrating on specific cancer types. The Python Reddit API Wrapper (PRAW) library was used to retrieve posts published between January 2015 and January 2025 through keyword searches. The search terms included “radiotherapy,” “radiation,” “IMRT,” and “brachytherapy.” This process yielded an initial dataset comprising 52,831 records.

### Inclusion and Exclusion Criteria and Data Preprocessing

To ensure the relevance and high quality of the analyzed data, a rigorous screening process was implemented. Initially, a total of 4048 duplicate records were eliminated. Subsequently, the remaining 48,783 posts underwent preliminary cleaning, with the exclusion of 1562 non-English posts and 8547 posts deemed unsuitable for effective semantic analysis due to insufficient text length (<20 words). Subsequently, a team of 2 researchers with oncology backgrounds conducted a comprehensive review of the remaining 38,674 posts. The initial dataset contained 20,419 nonpatient first-person narratives, including inquiries from family members or friends, news links, and commercial advertisements. These were excluded from the analysis. In addition, 8395 posts discussing purely technical issues, such as device specifications, were excluded if they did not express personal experiences or emotional distress. Following this screening, a total of 9860 high-quality patient narrative posts were ultimately included in the final analysis.

Prior to the analysis, all texts underwent a series of standard preprocessing steps. This included the removal of URLs, special characters, and emoticons. The texts were then converted to lowercase and lemmatized, a process that merges words sharing the same root (eg, “anxious” and “anxiety” were processed as “anxiety”). In the interest of protecting privacy, all potentially identifiable information (eg, usernames and place names) was removed.

### Sample Characterization

Given the anonymous nature of Reddit, traditional demographic data (eg, verified age, gender, and geographic location) were not systematically available. To provide contextual characterization of the study sample, we conducted a systematic extraction of self-disclosed information from post content. A keyword-based extraction algorithm was developed to identify mentions of cancer type (eg, “breast cancer,” “head and neck,” and “prostate”), treatment phase (eg, “starting radiation next wk,” “just finished my last session,” and “3 mo post-treatment”), treatment intent (eg, “curative,” “palliative,” and “adjuvant”), gender (eg, explicit statements, such as “as a woman,” or gendered pronouns in a self-referential context), and age (eg, “I’m 52” and “diagnosed at 38”). The source subreddit for each post was also recorded.

To assess the accuracy of automated extraction, a random 10% subsample (n=986) was independently reviewed by 2 researchers. Agreement between automated extraction and manual verification was high for cancer type (κ=0.89) and treatment phase (κ=0.85), moderate for gender (κ=0.78), and low for age (κ=0.71) due to varied expression patterns. Discrepancies were resolved in favor of the manual classification. Characteristics were coded as “not identifiable” when posts did not contain relevant self-disclosures.

### Data Analysis

#### Topic Identification and Classification

The analytic workflow proceeded in 2 strictly sequential phases to minimize interpretive bias.

In the first phase (unsupervised topic discovery), latent Dirichlet allocation (LDA) topic modeling was applied to the full corpus of 9860 preprocessed posts without any prespecified classification framework. The model was implemented using the Gensim library (version 4.3.1) in Python 3.9. The algorithm operated purely on statistical word co-occurrence patterns to identify latent semantic themes, with no constraints imposed to align topics with predefined distress categories.

To determine the optimal number of topics (k), we systematically evaluated candidate models ranging from k=5 to k=30 (increments of 1). For each candidate, we computed the C_v coherence score—a metric that measures semantic interpretability based on word co-occurrence patterns—and log perplexity. The coherence score reached its maximum at k=17 (C_v=0.52), beyond which values plateaued and showed marginal decline (see Multimedia Appendix 1 for the coherence score curve). Additionally, qualitative inspection of topics at k=15, 16, 17, 18, and 19 was performed by the research team. Models with fewer topics merged conceptually distinct themes (eg, claustrophobia and skin reactions collapsed into a single topic), while models with more topics produced redundant or incoherent themes. Based on the convergence of quantitative metrics and qualitative interpretability, k=17 was selected.

The following hyperparameters were used: α (document-topic density) = “auto” (asymmetric prior; optimized during model training); β/eta (topic-word density) = “auto” (symmetric prior; optimized during training); number of passes over the full corpus=50; chunk size=2000 documents; and random state seed=42 for reproducibility. Full parameter specifications are provided in Multimedia Appendix 2.

The trained LDA model outputs a probability distribution over all 17 topics for each post. For downstream analyses (prevalence estimation, correlation analysis, and sentiment analysis), each post was assigned to its dominant topic—defined as the topic with the highest posterior probability. All 9860 posts received a dominant topic assignment and were included in the theme-level analyses. The 0.30 probability threshold was used during model development as a quality benchmark to assess assignment confidence. Qualitative review of posts near this boundary confirmed that dominant-topic assignments remained interpretable, and no posts were excluded from theme-level analyses on this basis. This phase ultimately yielded 17 topics, each represented by a set of high-probability keywords.

In the second phase (post-hoc qualitative interpretation and classification), 2 researchers with oncology backgrounds independently reviewed each topic’s top 30 keywords and a minimum of 50 representative original posts to assign a descriptive thematic label (eg, “fear of recurrence and scanxiety”). Importantly, this naming process was completed before classification was attempted.

Only after all 17 themes were named did the 2 researchers independently classify each theme into one of two categories based on the following operational definitions:

1. General cancer distress: Psychosocial issues related to the universal impacts of a cancer diagnosis, prognosis, or treatment that are not specific to any single treatment modality (eg, fear of recurrence, social isolation, and existential concerns) [13].
2. Radiotherapy-specific distress: Psychosocial issues directly arising from the unique processes, equipment, environment, or acute/late side effects that are exclusive to radiation therapy (eg, immobilization-related claustrophobia, radiation skin burns, and daily treatment logistics) [14].

Initial independent classification yielded agreement on 16 of 17 topics (Cohen κ=0.92). The single discrepant case—topic B8 (“post-radiation blues and end-of-treatment anxiety”)—was initially classified as general distress by 1 researcher (reasoning that end-of-treatment anxiety is common across modalities) and as radiotherapy-specific distress by the other (reasoning that it was triggered by the abrupt cessation of daily radiation sessions). After joint re-examination of 50 representative posts for this topic, both researchers agreed that the narratives predominantly referenced the loss of the daily radiotherapy routine and the radiation team specifically, rather than generic treatment completion. The topic was therefore classified as radiotherapy-specific. No classification criteria were preregistered, and this constitutes a limitation discussed below. Full parameter specifications are provided in Multimedia Appendix 2.

We acknowledge that independent external validation of the classification by additional reviewers outside the research team was not performed. The operational definitions and classification outcomes therefore reflect the interpretive judgment of the 2 oncology-trained researchers and should be considered within this context. Future studies may benefit from involving independent external coders or using formal Delphi consensus procedures to strengthen classification validity.

#### Interthematic Correlation Analysis

A quantitative analysis of co-occurrence relationships across 17 themes was conducted to examine the co-occurrence patterns and thematic differentiation of the 2 types of distress. A posttheme matrix (9860×17) was constructed directly from the LDA output, where each cell value represents the posterior probability (ranging from 0 to 1) of a given topic within a given post. This continuous probability matrix—rather than the binary dominant-topic assignments used for prevalence estimation—was used for the correlation analysis to preserve the full distributional information of topic co-occurrence. The Pearson correlation coefficient was then calculated for each pair of the 17 topics based on their probability vectors across all posts. This coefficient is indicative of the frequency and strength of their simultaneous occurrence within the same set of posts. Finally, the 17×17 correlation matrix was represented graphically using a heatmap, where color intensity visually represents the strength of the correlation.

The heatmap displays the Pearson correlation coefficients for the co-occurrence of the 17 identified distress themes across 9860 patient posts. Color intensity corresponds to correlation strength: deep red indicates strong positive correlation (*r*≥0.60), orange indicates moderate correlation (0.30≤*r*<0.60), and yellow indicates weak or negligible correlation (*r*<0.30). Notable within-category correlations included A1-A4 (*r*=0.58), B1-B6 (*r*=0.70), and B2-B4 (*r*=0.52). Cross-category correlations were uniformly low (mean *r*=0.08; range: −0.05 to 0.18), visually represented by the lighter-colored off-diagonal quadrants. The diagonal is set to 1.0 (self-correlation). This pattern is consistent with thematic differentiation between the 2 researcher-assigned categories, though it should be interpreted as descriptive rather than confirmatory evidence (see Methods for a discussion of analytic limitations).

#### Sentiment and Emotion Analysis

In order to explore the differences in emotional expression between the 2 categories of distress, this study conducted an analysis at 2 levels: overall sentiment polarity and discrete emotion classification. At the first level, the VADER (Valence Aware Dictionary and Sentiment Reasoner) tool was used to calculate the compound sentiment score for each post. VADER is a dictionary- and rule-based sentiment analysis tool particularly suited for social media text, with compound scores ranging from −1 (most negative) to +1 (most positive) [15]. At the second level, discrete emotion classification was performed using the publicly available RoBERTa-base model fine-tuned on the GoEmotions dataset (model checkpoint: SamLowe/roberta-base-go_emotions; Hugging Face Model Hub) [16]. This model was selected for its domain congruence (trained on approximately 58,000 Reddit comments), taxonomic granularity (27 emotion categories, including “nervousness” as distinct from “fear”), and public reproducibility. The 27 GoEmotions categories were mapped to 4 clinically relevant negative emotion supercategories: “anxiety” (nervousness), “fear” (fear), “sadness” (sadness plus grief), and “anger/frustration” (anger plus annoyance). The primary emotion per post was defined as the supercategory with the highest summed probability score. Posts with maximum scores below 0.15 (n=287, 2.9%) were classified as “emotion indeterminate” and excluded from the emotion-type distribution analysis.

It should be noted that the RoBERTa-based emotion classifier was trained on general Reddit comments (GoEmotions dataset) rather than oncology-specific narratives. While this provides partial domain congruence given our Reddit-sourced data, certain emotion categories may perform differently in health-related contexts. For example, expressions of treatment-related suffering (eg, “this is torture”) might be classified as “anger” rather than “fear” due to lexical patterns in the training data that associate such phrasing with frustration rather than threat appraisal. No manual validation of model outputs against expert annotation was performed on a subset of the present corpus. The emotion classification results should therefore be interpreted with this caveat in mind. Full model specifications, performance metrics, the complete category mapping rationale, and exclusion details are provided in Multimedia Appendix 3.

### Statistical Analysis

All statistical analyses were performed using Python 3.9 (SciPy version 1.11.2, Statsmodels version 0.14.0). The significance level was set at *α*=.05 (2-tailed) for all tests.

For the comparison of VADER compound scores between the 2 distress categories, the Mann-Whitney *U* test was adopted as the sole primary inferential test. This choice was made on the following theoretical grounds: (1) VADER compound scores are bounded between −1 and +1, which precludes a true normal distribution—a Gaussian distribution requires unbounded support on (−∞, +∞) [15]; (2) a corpus composed exclusively of distress-related narratives is expected to be negatively skewed a priori, as posts overwhelmingly express negative rather than positive sentiment; and (3) the Mann-Whitney *U* test makes no distributional assumptions and is appropriate for comparing the location shift of 2 independent samples under these conditions. The rank-biserial correlation (r_rb) was computed as the primary effect size measure for this test, calculated as r_rb=1 − (2U)/(n₁ × n₂), with values interpreted as small (0.10), medium (0.30), and large (0.50). Cohen *d* was additionally computed from the group means and pooled SD as a supplementary standardized mean difference measure to facilitate comparison with published literature.

The chi-square test of independence was used to examine differences in the distribution of primary negative emotions (anxiety, fear, sadness, and anger/frustration) between the 2 groups. Cramér V was computed as the corresponding effect size, calculated as V = √(χ²/(n × (min(r,c) − 1))), with values interpreted as small (0.10), medium (0.30), and large (0.50) following Cohen conventions.

We acknowledge that given the large sample sizes (n>4800 per group), the Central Limit Theorem ensures that the sampling distribution of the mean approximates normality regardless of the underlying distribution shape, rendering parametric tests theoretically applicable. The independent samples *t* test was therefore conducted as a supplementary sensitivity analysis to confirm the robustness of conclusions to analytic choice, and its results are reported alongside the primary nonparametric findings.

### Ethical Considerations

The data used in this study were obtained exclusively from publicly accessible online platforms where users voluntarily shared information. During the course of data collection and analysis, strict adherence was observed to the terms of the platforms’ end-user agreements. Prior to analysis, all data underwent rigorous anonymization to ensure the absence of any traceable links to individual identities, thereby safeguarding user privacy. It should be noted that this research did not involve direct interaction with human subjects and complies with the ethical guidelines for using publicly available data outlined in the Declaration of Helsinki.

## Results

### Sample Description and Screening Process

**Figure 1.**
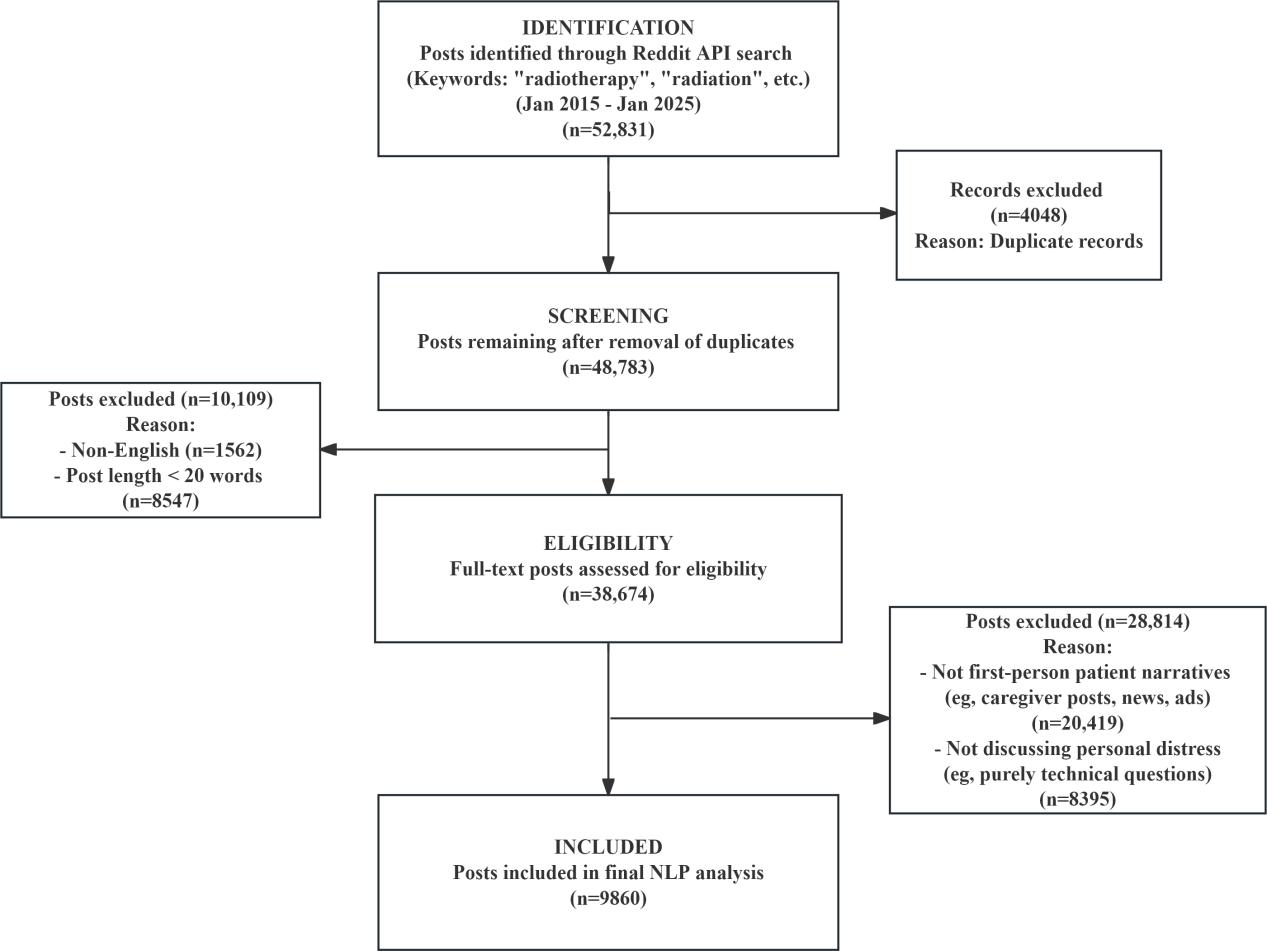
Flow diagram of the study selection process. API: application programming interface; NLP: natural language processing.

The data screening process for this study is illustrated in a study flow diagram (Figure 1), which has been structured to parallel the transparency principles of systematic reporting frameworks. A preliminary investigation was conducted to ascertain the number of relevant Reddit posts. A total of 52,831 relevant posts were identified through keyword searches on the Reddit social media platform, spanning from January 2015 to January 2025. Following the elimination of 4048 duplicate records, the remaining 48,783 posts advanced to the screening phase. Subsequently, 1562 non-English posts and 8547 posts with text lengths under 20 words were excluded. During the eligibility assessment phase, a full-text review of the remaining 38,674 posts was conducted. This resulted in the exclusion of 20,419 nonpatient first-person narratives (eg, family inquiries, news articles, or advertisements) and 8395 posts that discussed purely technical issues without expressing personal distress. The final sample comprised 9860 patient narratives that met the specified criteria, which were then subjected to an NLP analysis. All 9860 posts subjected to LDA topic modeling received a dominant topic assignment and were included in theme-level prevalence, correlation, and sentiment analyses. Among these, 4969 (50.4%) posts were classified under radiotherapy-specific distress themes, 4831 (49.0%) posts were classified under general cancer distress themes, and 60 (0.6%) posts could not be unambiguously classified into either category based on the operational definitions.

### Sample Characteristics

The contextual characteristics extractable from the 9860 included posts are summarized in Table 1. Cancer type was identifiable in 67.2% (6625/9860) of posts. The 3 most frequently mentioned cancer types were head and neck cancer (1576/6625, 23.8%), breast cancer (1418/6625, 21.4%), and prostate cancer (1000/6625, 15.1%), followed by lung cancer (643/6625, 9.7%), cervical/gynecological cancers (550/6625, 8.3%), and brain tumors (411/6625, 6.2%). Other or rarer cancer types accounted for 15.5% (1027/6625) of cases. This distribution is consistent with cancer types for which radiotherapy is a primary or adjuvant treatment component, providing face validity for the sample’s relevance to the study question.

**Table 1.** Contextual characteristics extracted from included patient narratives (N=9860).

Characteristic	Distribution, n (%)
Cancer type	6625 (67.2)
Head and neck	1576 (23.8)
Breast	1418 (21.4)
Prostate	1000 (15.1)
Lung	643 (9.7)
Cervical/gynecological	550 (8.3)
Brain	411 (6.2)
Other/multiple	1027 (15.5)
Treatment phase	5748 (58.3)
During active radiotherapy	3587 (62.4)
Early posttreatment (≤3 months)	1420 (24.7)
Long-term survivorship (>3 months)	741 (12.9)
Treatment intent	4092 (41.5)
Curative	3217 (78.6)
Palliative	875 (21.4)
Gender	4397 (44.6)
Female	2300 (52.3)
Male	2097 (47.7)
Age group	1863 (18.9)
18‐39 years	412 (22.1)
40‐64 years	1093 (58.7)
≥65 years	358 (19.2)
Source subreddit	9860 (100)
r/cancer	4171 (42.3)
r/RadiationTherapy	1844 (18.7)
Cancer type–specific subreddits	2800 (28.4)
Other health communities	1045 (10.6)

The treatment phase was identifiable in 58.3% (5748/9860) of posts. Among these, 62.4% (3587/5748) were written during active radiotherapy, 24.7% (1420/5748) were written during the early posttreatment period (within 3 months of completion), and 12.9% (741/5748) were written during long-term survivorship (>3 months posttreatment). Treatment intent was identifiable in 41.5% (4092/9860) of posts, with curative intent predominating (3217/4092, 78.6%) over palliative intent (875/4092, 21.4%).

Gender was inferable in 44.6% (4397/9860) of posts, with a relatively balanced distribution: 52.3% (2300/4397) female and 47.7% (2097/4397) male. Age information was explicitly stated in only 18.9% (1863/9860) of posts, and among those, the distribution was as follows: 18‐39 years (412/1863, 22.1%), 40‐64 years (1093/1863, 58.7%), and ≥65 years (358/1863, 19.2%).

Regarding source subreddits, posts originated primarily from r/cancer (4171/9860, 42.3%), r/RadiationTherapy (1844/9860, 18.7%), cancer type-specific subreddits (eg, r/breastcancer and r/prostatecancer; 2800/9860, 28.4%), and other health-related communities (1045/9860, 10.6%).

### Identification and Overall Distribution of Psychological Distress Themes

The use of thematic modeling and manual qualitative coding resulted in the identification of 17 core psychological distress themes from a total of 9860 posts (see Table 2). These themes were subsequently categorized into 2 broad classifications with consideration of distress: “general cancer distress” and “radiotherapy-specific distress.”

**Table 2.** Identified psychological distress themes, definitions, and prevalence in radiotherapy patient narratives (9860 posts).

Theme number	Topic name	Core keywords	Representative quote	Category	Prevalence (N=9860), n (%)
A1	Fear of recurrence and scanxiety	scan, scanxiety, recurrence, worried, waiting, results, ache, fear, anxious	My 6-month scan is next week and I’m a wreck. Every little ache sends me into a spiral, thinking “Is it back?.” The waiting is a special kind of hell.	General	1390 (14.1)
B1	Claustrophobia and panic from immobilization devices	mask, mold, bolted, trapped, panic, claustrophobic, breathe, terror, Ativan	The moment they bolt the mask down, I have a full-blown panic attack. It feels like being buried alive. Chemo was poison, but this is mental torture.	Radiotherapy-specific	1133 (11.5)
B2	Acute skin reactions: pain, burning and body image	skin, burn, red, peeling, lotion, pain, Aquaphor, sore, disfigured	My skin is on fire. It’s bright red, weeping, and even a soft t-shirt is agony. I look in the mirror and just feel so damaged and ugly.	Radiotherapy-specific	907 (9.2)
A2	Financial toxicity and insurance hassles	cost, insurance, bills, work, afford, debt, copay, financial, stress	Honestly, I’m more scared of the medical bills than the cancer itself. How are we supposed to afford this even with insurance?	General	818 (8.3)
B3	Logistical burden and daily treatment fatigue	daily, drive, travel, tired, exhausting, schedule, every day, burnout	It’s the relentless daily trips to the hospital that are draining my soul. My life has shrunk to my house and the radiation center.	Radiotherapy-specific	759 (7.7)
A3	Social isolation and misunderstanding	friends, family, alone, nobody understands, lonely, disconnected, isolate	My friends want the ‘old me’ back, but she’s gone. I feel so alone in this, even when I’m in a crowded room.	General	680 (6.9)
B4	Mucositis and swallowing difficulties	throat, mouth sores, swallow, pain, eating, magic mouthwash, feeding tube	It feels like swallowing glass. I’ve lost 20 pounds because I just can’t eat. The pain is unbearable.	Radiotherapy-specific	631 (6.4)
A4	Posttreatment identity crisis and depression	lost, empty, after, new normal, survivor, depressed, purpose, sadness	I’m officially NED, but I feel completely empty. Who am I now that I’m not ‘the patient’ anymore?	General	562 (5.7)
A5	Impact on family roles and caregiving	husband, wife, kids, burden, partner, guilt, parent, relationship	I feel so guilty that my husband has become my full-time caregiver. It’s put such a strain on our relationship.	General	512 (5.2)
B5	Fear of radiation and misconceptions	radiation, radioactive, safety, exposure, glowing, danger, kids, pets	A genuine question: Am I radioactive after treatment? Is it safe for me to hold my grandkids or sleep in the same bed as my partner?	Radiotherapy-specific	423 (4.3)
A6	Existential dread and mortality anxiety	dying, death, fear, future, legacy, spiritual, meaning, scared	Facing my own mortality at 40 is something I never prepared for. It keeps me up at night, just thinking about the ‘what ifs.’	General	404 (4.1)
B7	Bowel/bladder dysfunction from pelvic radiotherapy	diarrhea, bathroom, urgency, bladder, bowel, incontinence, diet, cramps	My life now revolves around being within 10 feet of a bathroom. I can’t go out. The urgency and cramping are constant.	Radiotherapy-specific	345 (3.5)
B8	Postradiation blues and end-of-treatment anxiety	last day, finished, now what, bell, lost, abandoned, safety net, anxious	I rang the bell today, but I don’t feel happy. I feel... abandoned. The daily safety net of seeing the team is gone.	Radiotherapy-specific	306 (3.1)
A8	Navigating the health care system	doctors, appointments, waiting, communication, team, confused, overwhelmed	I have five different doctors and none of them seem to talk to each other. I’m so overwhelmed trying to coordinate my own care.	General	277 (2.8)
B6	Isolation and coldness of the treatment environment	alone, machine, cold, sterile, bunker, impersonal, loud, humming	Lying alone in that cold, sterile room with that giant machine humming over me is one of the most isolating experiences of my life.	Radiotherapy-specific	257 (2.6)
B9	Fear of long-term side effects	long-term, fibrosis, permanent, damage, secondary cancer, years later	My RO mentioned fibrosis and the risk of secondary cancers. I’m worried that the ‘cure’ will cause new problems 10 years from now.	Radiotherapy-specific	208 (2.1)
A7	Career disruption and disability concerns	job, career, disability, return to work, boss, income, FMLA^a^	How am I supposed to go back to my demanding job? I have brain fog and fatigue. Will my boss even understand?	General	188 (1.9)
—^b^	Other/unclassified	—	—	—	60 (0.6)

a FMLA: Family and Medical Leave Act.

b Not applicable.

Radiotherapy-specific distress prevailed in the patients’ narratives. In the corpus of posts addressing psychological distress, radiotherapy-specific distress constituted 50.4% (4969/9860) of posts, marginally surpassing general cancer distress, which accounted for 49.0% (4831/9860) of posts. The remaining 0.6% (60/9860) of posts were classified as “other” or “unclassifiable.” This finding indicates that challenges unique to radiotherapy constitute a component of patients’ overall psychological burden that is nearly as significant as cancer itself.

Table 2 presents a comprehensive list of the 17 themes, including their respective names, core keywords, representative quotes, category, and occurrence frequency within the total sample. Among all themes, “fear of recurrence and scanxiety (A1)” was the most prevalent general concern, accounting for 14.1% (1390/9860) of all posts. One patient articulated their sentiments with the following written statement:

My 6-month scan is next week, and I’m an absolute mess. Every single little ache or pain sends me spiraling into panic, thinking, ‘Is it coming back?’ The waiting is just this unique kind of torture.

However, among radiotherapy-specific concerns, “claustrophobia and panic from immobilization devices (B1)” emerged as the most prominent issue, accounting for 11.5% (1133/9860) of posts. The intensity and distinctiveness of the phenomenon under study were vividly captured in patient narratives:

I get so anxious and panicky when they strap that mask on tight. It feels like being buried alive. Chemo is poison, but radiation is pure mental torture.

Following closely were “acute skin reactions (B2)” (907/9860, 9.2%) and “daily treatment logistical burden (B3)” (759/9860, 7.7%), both of which are distinctive sources of suffering directly stemming from the radiotherapy process.

### Structural Relationships Among Themes: Internal Cohesion and External Differentiation

**Figure 2.**
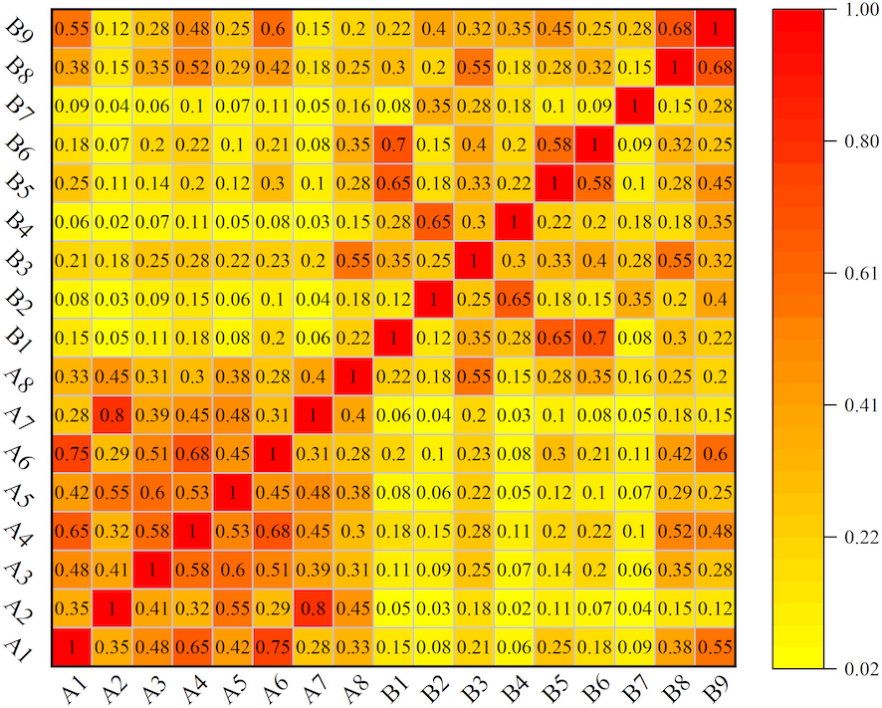
Correlation heatmap of psychological distress themes. A1: fear of recurrence and scanxiety; A2: financial toxicity and insurance hassles; A3: social isolation and misunderstanding; A4: posttreatment identity crisis and depression; A5: impact on family roles and caregiving; A6: existential dread and mortality anxiety; A7: career disruption and disability concerns; A8: navigating the health care system; B1: claustrophobia and panic from immobilization devices; B2: acute skin reactions: pain, burning and body image; B3: logistical burden and daily treatment fatigue; B4: mucositis and swallowing difficulties; B5: fear of radiation and misconceptions; B6: isolation and coldness of the treatment environment; B7: bowel/bladder dysfunction from pelvic radiation therapy; B8: postradiation blues and end-of-treatment anxiety; B9: fear of long-term side effects.

To explore whether the 2 researcher-assigned categories exhibit distinct co-occurrence patterns, we examined the correlational relationships among the 17 themes. It should be noted that this analysis is descriptive rather than an independent validation of the classification framework, as both the thematic structure and the category assignments derive from the same analytic pipeline. Accordingly, the results are interpreted as evidence consistent with thematic differentiation rather than confirmation of structural independence. The results of this study are presented in the heatmap shown in Figure 2. The intensity of the colors in the figure is indicative of the strength of the correlation between the themes. Red is used to indicate a strong correlation, while yellow is used to indicate a weak correlation. The results of the study manifest in the following two key patterns:

1. Internal cohesion: Within the lower-left quadrant, which represents “general cancer distress” (A1-A8), and the upper-right quadrant, which represents “radiotherapy-specific distress” (B1-B9), extensive areas of deep red and orange appear. This finding suggests the presence of robust co-occurrence relationships among themes within each respective category. For instance, the fear of recurrence (A1) was frequently mentioned in conjunction with social isolation (A3) (*r*=0.48) and posttreatment identity crisis (A4) (*r*=0.58). Similarly, “claustrophobia (B1)” exhibited a substantial correlation with “coldness of the treatment environment (B6)” (*r*=0.70).
2. Low cross-category association: In contrast, the cross-over regions connecting the 2 major thematic categories (upper left and lower right corners) exhibit predominantly lighter colors (yellow), indicating weak co-occurrence between general cancer distress and radiotherapy-specific distress themes. This pattern is consistent with thematic differentiation between the 2 researcher-assigned categories, though it does not constitute independent proof of structural independence.

This correlational pattern is consistent with the interpretation that general cancer distress themes and radiotherapy-specific distress themes form 2 thematically differentiable clusters with high within-cluster cohesion and low between-cluster association. However, because the category assignments were made by the same researchers who interpreted the LDA output, this analysis should be regarded as descriptive support for the proposed dual-domain framework rather than independent statistical confirmation of structural independence.

### Differential Analysis of Emotional Characteristics Between the Types of Distress

As summarized in Table 3, the sentiment score distribution for radiotherapy-specific distress posts was significantly shifted toward greater negativity compared with general cancer distress posts. The Mann-Whitney *U* test indicated a statistically significant difference between the 2 groups (median: −0.58 vs −0.32; *U*=7,921,729; *P*<.001). The rank-biserial correlation was r_rb=0.34, indicating a medium effect size, and this indicates that in approximately 67% of randomly drawn pairs (1 post from each group), the radiotherapy-specific post would exhibit more negative sentiment. The mean scores reflected a consistent pattern (mean −0.52, SD 0.28 vs mean −0.35, SD 0.21; Cohen *d*=0.69), corresponding to a medium-to-large standardized difference. A sensitivity analysis using the independent samples *t* test confirmed the robustness of this finding (*t*_9798_=−15.72, *P*<.001).

**Table 3.** Comparison of sentiment scores between general cancer distress and radiotherapy-specific distress.

Measure	General cancer distress (n=4831)	Radiotherapy-specific distress (n=4969)	*t* test^a^ (*df*)	*U* value	*P* value	Effect size^b^^,^^c^
VADER^d^ compound score^e^^,^^f^						
Mean (SD)	−0.35 (0.21)	−0.52 (0.28)	−15.72 (9798)	—^g^	<.001	Cohen *d*=0.69
Median (IQR)	−0.32 (−0.45, −0.20)	−0.58 (−0.75, −0.30)	—	7,921,729	<.001	r_rb=0.34

a An independent samples *t*-test was conducted as a sensitivity analysis and yielded consistent conclusions, confirming robustness to analytic choice.

b Cohen *d* was computed from group means and pooled SDs. Rank-biserial correlation (r_rb) was computed from the Mann-Whitney *U* statistic using the formula r_rb = 1 − (2U) / (n₁ × n₂).

c Rank-biserial correlation (r_rb): small=0.10, medium=0.30, large=0.50; Cohen *d*: small=0.20, medium=0.50, large=0.80.

d VADER: Valence Aware Dictionary and Sentiment Reasoner.

e VADER compound scores range from −1 (most negative) to +1 (most positive). Both distributions significantly deviated from normality (Shapiro-Wilk *P*<.001); therefore, the Mann-Whitney *U* test was adopted as the primary comparison.

f For VADER compound score comparisons, all posts classified under each distress category were included (n=4831 and n=4969).

g Not applicable.

Beyond overall negative intensity, a clinically meaningful divergence emerged in the distribution of primary emotion types (Table 4). The chi-square test confirmed a significant difference in the distribution of dominant negative emotions between the 2 groups (*χ*²_3_=234.56; *P*<.001; Cramér V=0.22), representing a small-to-medium effect. General cancer distress was predominantly characterized by chronic, diffuse emotional states—anxiety (2183/4831, 45.2%) and sadness (1599/4831, 33.1%)—whereas radiotherapy-specific distress was dominated by acute, event-triggered emotions—fear (2773/4969, 55.8%) and anger/frustration (1262/4969, 25.4%). This emotional signature—where acute fear and anger are triggered by specific, identifiable treatment events (eg, immobilization mask application and acute radiation burns)—reveals a qualitatively distinct pattern of psychological distress from that of general cancer anxiety and suggests the need for fundamentally different intervention approaches.

**Table 4.** Comparison of emotional profiles between general cancer distress and radiotherapy-specific distress.

Measure	General cancer distress (n=4831), n (%)	Radiotherapy-specific distress (n=4969), n (%)	Chi-square (*df*)	*P* value	Effect size^a^
Primary negative emotion^b^^,^^c^			234.56 (3)	<.001	Cramér V=0.22
Anxiety	2183 (5.2)	760 (15.3)			
Sadness	1599 (33.1)	174 (3.5)			
Fear	894 (18.5)	2773 (55.8)			
Anger/frustration	155 (3.2)	1262 (25.4)			

a Cramér V: small=0.10, medium=0.30, large=0.50.

b Emotion classification was performed using the RoBERTa-based model. Percentages represent the proportion of posts where each emotion was identified as the primary (highest probability) emotion.

c For primary negative emotion distribution, 287 (2.9%) posts classified as "emotion indeterminate" were excluded; the percentages shown are calculated within each group's remaining posts with identifiable primary emotions.

## Discussion

### Principal Findings

This study, through an analysis of a large-scale corpus of patient narratives, prompts us to re-examine a phenomenon potentially underestimated in clinical practice: the specific psychological distress directly induced by radiotherapy appears to hold nearly equal prominence to general cancer distress within patients’ online narratives. While our exploratory findings do not establish definitive psychological independence between these domains, the observed thematic and emotional differentiation suggests that conventional psychosocial support models characterized by a pan-cancer perspective may benefit from incorporating treatment-specific considerations. When assessing and intervening in the mental health of patients undergoing radiotherapy, the unique sources of distress generated by the treatment process itself warrant clinical attention alongside general cancer concerns.

These findings extend existing psychosocial oncology literature. Prior studies have focused primarily on concerns, such as fear of recurrence and social disconnection [17-19]. Our analysis confirms that these remain fundamental patient concerns but further reveals that patients’ psychological distress is not characterized by a single, generalized “cancer anxiety” alone. This study presents exploratory evidence for a dual-distress thematic framework: one cluster of themes concerns the existential and social challenges of “being a patient with cancer,” while another equally prominent cluster stems from the specific, procedural physical and mental experience of “undergoing radiation therapy.”

It is important to contextualize these findings within the characteristics of the study sample. The prominence of claustrophobia from immobilization devices (B1) as the leading radiotherapy-specific concern likely reflects the high representation of patients with head and neck cancer (23.8%) in our corpus—a population for whom thermoplastic mask immobilization is a routine and often distressing component of treatment. Samples with different cancer-type compositions (eg, predominantly pelvic cancers) might yield different rankings of radiotherapy-specific themes, potentially elevating bowel/bladder dysfunction (B7) to greater prominence. Similarly, the relatively balanced gender distribution observed in our identifiable subsample (52.3% female, 47.7% male) may explain the absence of a strongly gender-specific theme. In more homogeneous clinical samples, themes, such as sexual dysfunction or fertility concerns following pelvic radiation, might emerge more distinctly. These observations underscore that the 17-theme framework identified here represents the distress landscape of a specific online community and requires cross-validation in diverse clinical populations.

The distinctiveness of radiotherapy-specific distress stems from its unique, perceptible triggers. In contrast to the chronic, diffuse emotional states characterized by “anxiety” and “sadness” in general cancer distress [20], radiotherapy-specific distress manifests more as acute “fear” and “anger” triggered by specific events. For instance, the most prevalent specific distress in this study—“claustrophobia induced by fixation devices”—is characterized by intense, immediate panic. Consequently, the severe pain from “acute skin reactions” and “mucositis” and the exhaustion stemming from the “logistical burden of daily treatments” serve as concrete, tangible stressors. This qualitative observation is corroborated by quantitative findings from our emotional analysis, which revealed that radiotherapy-specific distress manifests with greater negative emotional intensity, with core emotions comprising “fear” and “anger/frustration.” This phenomenon is characterized by a distinct emotional profile, one that differs from the commonly observed feelings of anxiety and sadness associated with general cancer distress [21]. Notably, the observed effect sizes (rank-biserial correlation=0.34 for sentiment intensity; Cramér V=0.22 for emotion type distribution) indicate medium-magnitude differences. While statistically significant, these effect sizes suggest meaningful but not absolute separation between the 2 domains—a finding consistent with the clinical reality that patients simultaneously navigate both general cancer concerns and treatment-specific challenges, with neither domain entirely eclipsing the other. The practical significance of these differences lies not in their magnitude alone, but in their qualitative implications for intervention design: acute fear responses (as seen in radiotherapy-specific distress) typically require immediate, situation-specific coping strategies (eg, relaxation techniques during immobilization), whereas chronic anxiety and sadness (as seen in general cancer distress) may respond better to longer-term psychotherapeutic and supportive interventions.

The findings of this study may provide some guidance for oncology nursing practice. Although contemporary psychological screening instruments (eg, the National Comprehensive Cancer Network “Distress Thermometer” [22]) are adept at identifying the presence of distress, their “one-dimensional” scoring model frequently lacks the capacity to discern the underlying cause of distress. These findings support the implementation of a more sophisticated stratified assessment model that matches interventions to specific distress sources. For patients experiencing immobilization-related claustrophobia, pretreatment equipment familiarization sessions combined with breathing relaxation training may reduce anticipatory panic and improve tolerance of daily sessions. When acute skin reactions emerge as a primary concern, nursing care should extend beyond passive symptom management to incorporate proactive body image counseling, acknowledging that visible radiation damage carries psychological weight beyond its physical discomfort. Similarly, the phenomenon of posttreatment emotional loss—whereby patients experience abandonment upon the abrupt cessation of daily radiotherapy contact—suggests that nursing teams should establish structured transition-phase protocols that gradually taper supportive contact rather than terminating it abruptly at treatment completion.

### Limitations

While this study used large-scale, unguided patient narrative data to offer unique ecological validity for understanding subjective patient experiences, the interpretation of its findings necessitates careful consideration of some limitations. First, although we extracted contextual characteristics from post content (Table 1), Reddit does not provide verified demographic data, and substantial proportions of posts lacked identifiable information for key variables (eg, age was extractable in only 18.9% of posts). The platform’s user base is known to be skewed toward younger individuals, male individuals, English-speaking individuals, and those having a higher educational attainment compared to the general population. This demographic profile likely shaped the observed thematic distribution in several ways. The relatively high proportion of posts from younger adults (22.1% aged 18‐39 years among those with identifiable age) may have amplified themes related to career disruption (A7) and social isolation (A3), which may be more salient for working-age individuals. Conversely, concerns more prevalent among older patients—such as comorbidity burden, caregiver dependence, and end-of-life decision-making—may be underrepresented in our corpus. The predominance of head and neck cancer (23.8%) and breast cancer (21.4%) in our sample, while consistent with cancers frequently treated with radiotherapy, indicates that specific distress experiences associated with less-represented cancers (eg, rectal cancer and pediatric cancers) may not be adequately captured. Furthermore, the requirement for English proficiency and digital literacy inherently excludes patients from non-English-speaking backgrounds and those with limited internet access, potentially underrepresenting culturally specific expressions of distress.

Additionally, a methodological limitation inherent to this study’s analytic design warrants acknowledgment. The classification of LDA-generated topics into “general cancer distress” and “radiotherapy-specific distress” was performed post hoc by the research team based on operational definitions that were not preregistered. Although the LDA was conducted blind to any classification scheme, the subsequent manual categorization introduces an interpretive layer that is inherently subjective. Furthermore, the correlation heatmap analysis used to examine the structural relationships between the 2 categories was derived from the same LDA posttheme matrix, meaning it cannot function as a fully independent validation of the dual-domain framework. The observed correlational pattern is therefore best interpreted as descriptive support consistent with the proposed classification, rather than definitive statistical proof of structural independence. Future research should seek to validate this dual-domain structure using independent datasets, externally derived classification criteria, or confirmatory approaches such as structural equation modeling applied to prospectively collected clinical data.

A further methodological limitation concerns the emotion classification pipeline. The RoBERTa model used for discrete emotion detection was fine-tuned on the GoEmotions dataset, which comprises general Reddit comments spanning diverse topics. Although this training corpus shares platform-level linguistic conventions with our data source, it was not specifically developed for or validated on oncology patient narratives. Health-related emotional expressions may carry domain-specific connotations that diverge from general conversational patterns. For instance, cancer patients’ descriptions of physical suffering may overlap lexically with expressions of anger or frustration in the training data, potentially leading to misclassification of pain-related distress. The absence of a manual validation substudy—in which expert annotators would independently classify emotions in a random sample of posts for comparison against model outputs—represents a limitation that future research should address. Specifically, we recommend that subsequent studies conduct such validation on a minimum of 200‐300 randomly sampled posts, reporting interrater reliability and model-human agreement metrics (eg, Cohen κ and *F*_1_-scores per emotion category). Despite this limitation, the consistent pattern of results across 2 independent analytic approaches (VADER for polarity and RoBERTa for discrete emotions) provides some convergent support for the observed emotional differences between the 2 distress categories.

Consequently, the generalizability of the identified thematic distributions to the clinical population as a whole should be approached with caution. Second, the cross-sectional design of this study captures distress states only at a specific point in time, without revealing the dynamic evolution of these psychological distresses throughout the radiotherapy process. Consequently, the findings emphasize “what exists” rather than “how it changes,” and inferring causal relationships exceeds the scope of this study’s design. Moreover, the analysis was constrained to English-language texts. This limitation restricts the applicability of the results to non-English cultural contexts. Additionally, it may overlook the profound influence of different cultures on expressions of distress and coping strategies.

The exclusion criteria applied during data preprocessing may also have shaped which patient experiences are represented in the final corpus. The removal of posts shorter than 20 words, while necessary for meaningful semantic analysis, may have excluded brief but emotionally intense expressions of distress (eg, “I can’t do this anymore” or “the mask is killing me”). Similarly, the restriction to first-person patient narratives indicates that distress observed and reported by caregivers—who may notice aspects of suffering that patients themselves do not articulate—is absent from this analysis. These selection effects should be considered when interpreting the relative prevalence of identified themes.

Future research should use prospective longitudinal designs across diverse clinical cohorts to validate the thematic structures and their dynamic shifts identified here, thereby addressing the study’s limitations in sample representativeness and causal inference. Concurrently, there is a need to develop and validate clinical screening tools capable of rapidly distinguishing between different sources of distress. Furthermore, targeted, culturally adaptive nursing interventions should be designed and evaluated for the specific forms of distress highlighted in this study (eg, claustrophobia and logistical burdens), with the aim of delivering precision psychosocial support for radiotherapy patients.

### Conclusion

This study used NLP techniques to systematically analyze large-scale online patient narratives, thereby constructing an exploratory psychological distress landscape for patients undergoing radiotherapy. The core finding suggests that procedural and physiological distress directly associated with the radiotherapy process coexists with existential and social distress stemming from the cancer diagnosis itself, appearing to occupy comparable prominence within patients’ online narratives. This dual thematic pattern is reflected not only in differentiated distress content but also in divergent emotional characteristics and co-occurrence patterns. General cancer distress primarily manifests as a chronic psychological state centered on “anxiety” and “sadness,” whereas radiotherapy-specific distress tends to manifest as acute emotional reactions, triggered by specific treatment events and dominated by “fear” and “anger.” This finding offers an exploratory but conceptually important addition to psychosocial oncology. When assessing patient distress, shifting from a singular “disease-centered” perspective to a “disease-treatment dual-centered” approach may better capture the full spectrum of patient experience. From a clinical perspective, this observation suggests the value of nursing interventions that extend beyond conventional approaches to generalized cancer anxiety, incorporating assessment tools and support strategies designed to identify and address challenges unique to radiotherapy, such as equipment-related fears, procedural fatigue, and specific physiological toxicities. However, these clinical recommendations remain hypothesis-generating and require prospective validation before implementation. In summary, this study provides an empirical foundation for constructing a more refined psychological support framework for patients undergoing radiotherapy while highlighting the potential role of the nursing profession in alleviating treatment-specific distress.

## Supplementary material

10.2196/100874 Multimedia Appendix 1 Topic model selection: coherence score curve.

10.2196/100874 Multimedia Appendix 2 Latent Dirichlet allocation model parameters and computational environment.

10.2196/100874 Multimedia Appendix 3 Specification of the RoBERTa emotion classification model.

## Abbreviations

LDA: latent Dirichlet allocation
NLP: natural language processing
PRAW: Python Reddit API Wrapper
STROBE: Strengthening the Reporting of Observational Studies in Epidemiology
VADER: Valence Aware Dictionary and Sentiment Reasoner
