# Supplementary material for: Differentiating Radiotherapy-Specific Distress From General Cancer Distress: Natural Language Processing Analysis of Patient Narratives
Source: JMIR Form Res. 2026 Jul 14;10:e100874. doi: 10.2196/100874 (PMC13367937; doi:10.2196/100874)
Supplement: Multimedia Appendix 1 [file formative-v10-e100874-s001.docx]

Multimedia Appendix 1. Topic Model Selection: Coherence Score Curve.


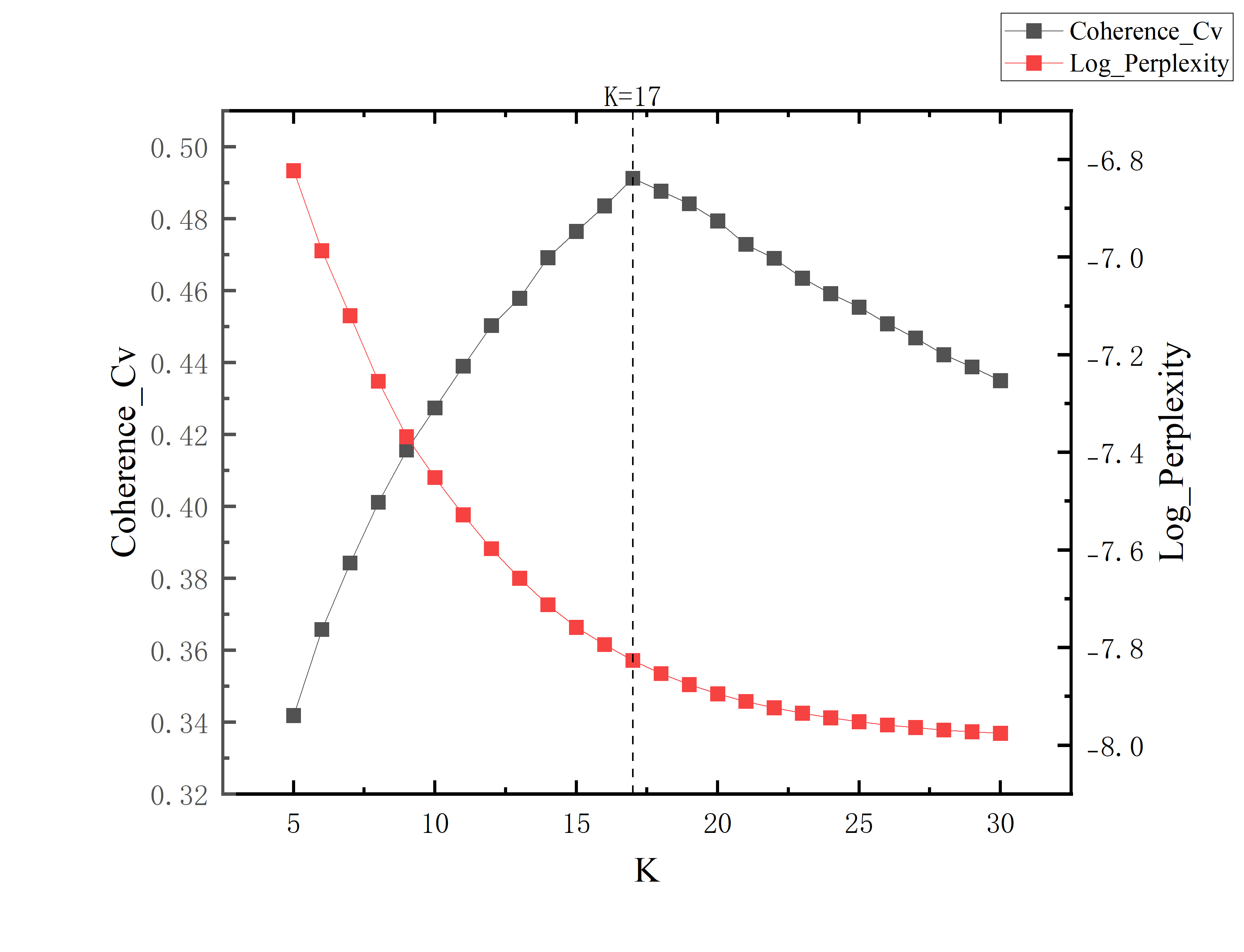
This figure displays the C_v coherence scores for LDA models with k = 5 to k = 30 topics. The x-axis represents the number of topics (k), and the y-axis represents the coherence score. The optimal model (k = 17, C_v = 0.52) is indicated by a vertical dashed line. The curve demonstrates a clear peak at k = 17, followed by a plateau and marginal decline, supporting the selection of this model.
