# Supplementary material for: Differentiating Radiotherapy-Specific Distress From General Cancer Distress: Natural Language Processing Analysis of Patient Narratives
Source: JMIR Form Res. 2026 Jul 14;10:e100874. doi: 10.2196/100874 (PMC13367937; doi:10.2196/100874)
Supplement: Multimedia Appendix 2 [file formative-v10-e100874-s002.docx]

Multimedia Appendix 2. LDA Model Parameters and Computational Environment.

| ***Parameter*** | ***Value*** |
| --- | --- |
| *Library* | *Gensim 4.3.1* |
| *Python version* | *3.9* |
| *Number of topics (k)* | *17* |
| *α (document-topic prior)* | *'auto' (asymmetric, optimized)* |
| *β / eta (topic-word prior)* | *'auto' (symmetric, optimized)* |
| *Number of passes* | *50* |
| *Chunk size* | *2,000* |
| *Random state* | *42* |
| *Minimum topic probability threshold for assignment* | *0.30* |
| *Evaluation metric for model selection* | *C_v coherence score* |
| *Optimal coherence score* | *0.52* |
| *Posts excluded due to sub-threshold assignment* | *312 (3.2%)* |
| *Preprocessing* | *Lowercasing, lemmatization, stop-word removal, URL/emoji removal* |
| *Minimum word frequency for inclusion in dictionary* | *15* |
| *Maximum document frequency for inclusion* | *0.50 (50% of corpus)* |
